# Intermolecular O–O Bond Formation between High-Valent Ru–oxo Species

**DOI:** 10.1021/acs.inorgchem.4c01560

**Published:** 2024-08-19

**Authors:** Tianqi Liu, Shaoqi Zhan, Biaobiao Zhang, Linqin Wang, Nannan Shen, Mårten S. G. Ahlquist, Xiaolei Fan, Licheng Sun

**Affiliations:** † Department of Chemistry, School of Engineering Sciences in Chemistry Biotechnology and Health, KTH Royal Institute of Technology, 10044 Stockholm, Sweden; ‡ Institute of Wenzhou, Zhejiang University, 325006 Wenzhou, China; § Department of Chemistry-BMC, Uppsala University, BMC Box 576, S-751 23 Uppsala, Sweden; ∥ Department of Chemistry—Ångström Laboratory; Uppsala University, Box 523, 75120 Uppsala, Sweden; ⊥ Center of Artificial Photosynthesis for Solar Fuels and Department of Chemistry, School of Science, Westlake University, 310024 Hangzhou, China; # State Key Laboratory of Radiation Medicine and Protection, School for Radiological and Interdisciplinary Sciences (RAD-X) and Collaborative Innovation Center of Radiation Medicine of Jiangsu Higher Education Institutions, Soochow University, 215123 Suzhou, China; ∇ Department of Chemical Engineering, School of Engineering, The University of Manchester, Oxford Road, Manchester M13 9PL, United Kingdom

## Abstract

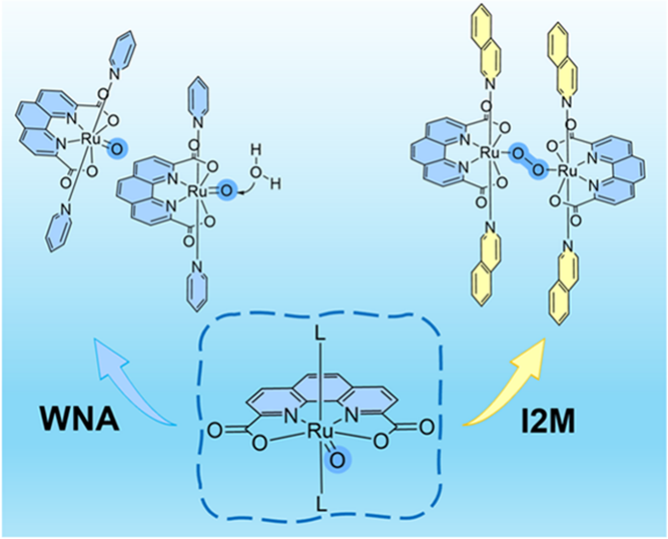

Despite extensive research on water oxidation catalysts over the past few decades, the relationship between high-valent metal-oxo intermediates and the O–O bond formation pathway has not been well clarified. Our previous study showed that the high spin density on O in Ru^V^=O is pivotal for the interaction of two metal-oxyl radical (I2M) pathways. In this study, we found that introducing an axially coordinating ligand, which is favorable for bimolecular coupling, into the Ru-pda catalyst can rearrange its geometry. The shifts in geometric orientation altered its O–O bond formation pathway from water nucleophilic attack (WNA) to I2M, resulting in a 70-fold increase in water oxidation activity. This implies that the I2M pathway is concurrently influenced by the spin density on oxo and the geometry organization of the catalysts. The observed mechanistic switch and theoretical studies provide insights into controlling reaction pathways for homogeneous water oxidation catalysis.

## Abstract 2

The impact of intermolecular directionality on water oxidation mechanisms was investigated by comparing the Ru-pda-type catalysts with pyridine and isoquinoline as axial ligands. It was clearly demonstrated that the intermolecular oxo–oxo coupling is simultaneously influenced by the spin density on the oxo group and the geometric arrangement of the catalysts.

## Introduction

One of the most important processes for producing sustainable energy is splitting water into oxygen, protons, and electrons.^1^ The extracted protons and electrons via water splitting can be used for hydrogen production, CO_2_ reduction, N_2_ reduction, etc.^2^ However, the overall water splitting efficiency was limited by the sluggish water oxidation reaction, which includes multielectron/proton transfer steps and O–O bond formation step.^3^ Particularly, the O–O bond formation step occupies 71% of the energy consumption of the total catalytic cycles of water oxidation, making it the rate-determining step (RDS) for the majority of water oxidation catalysts.^4^

Understanding this RDS can guide catalyst design with the aim of promoting the sluggish process of the water oxidation reaction. Typically, two main types of O–O bond formation mechanisms have been proposed, i.e., WNA^5−8^ and I2M,^9,10^ where the latter pathway is energetically preferable. Catalysts that operate via the I2M pathway, on the one hand, can provide lower onset potentials by avoiding the formation of high-energy metal-OOH intermediates.^4^ On the other hand, its turnover frequency (TOF) is catalyst-dependent, which allows for fast kinetics at the high catalyst concentration (TOF_I2M_ = *k* [cat], TOF_WNA_ = *k*).^11^ However, only limited occurrences of the intermolecular I2M pathway by Ru-based complexes have been recorded such far. For example, both the dinuclear Ru-aqua complex (where two aqua ligands are located at *trans* conformation) and the *cis*-[Ru(bpy)_2_(H_2_O)_2_]^2+^ displayed the second-order dependence on the catalyst concentration.^12,13^ In the latter one, the formation of a H-bond between the catalysts was proposed to promote the radical coupling pathway. Another example is the state-of-the-art Ru-bda (bda = 2,2-bipyridine-6,6-dicarboxylate) family water oxidation catalyst, which exhibited high activity comparable to that of photosystem II.^9,14,15^ Unfortunately, the discovery of catalysts with an I2M mechanism still relies heavily on serendipity. It is highly desirable and challenging to decipher the dominant factors behind the I2M mechanism to obtain conclusive guidelines for designing a larger amount of efficient catalysts based on the I2M pathway.^16,17^

**Figure 1:**
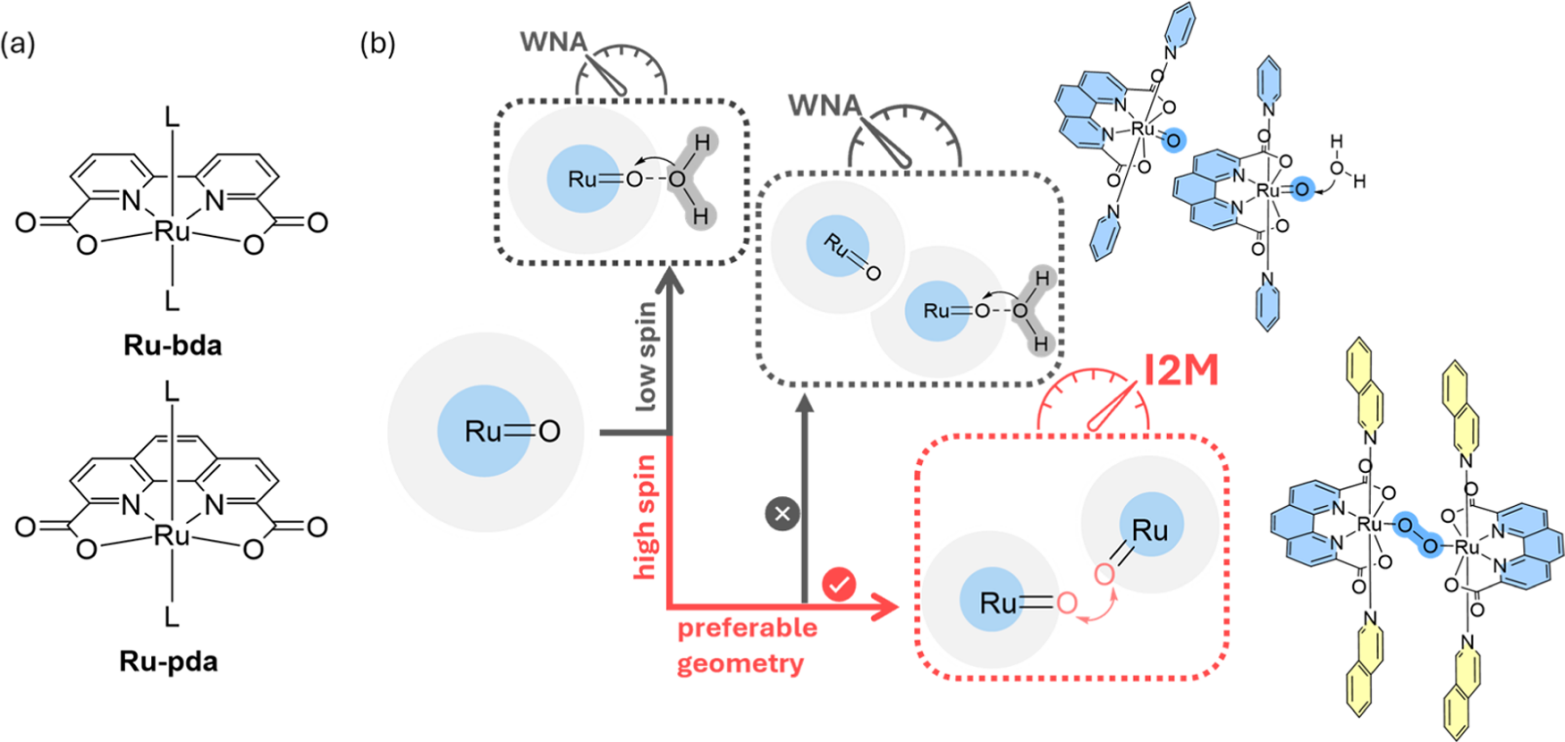
(a) Structures of water oxidation catalysts discussed in this paper; L is the axial ligand. (b) Schematic diagram of spin density and preferable geometry effects for the O–O bond formation mechanism.

Ru-bda is a well-studied water oxidation catalyst with an I2M mechanism. Our previous research revealed that hydrophobic Ru^V^(bda)=O with high spin density on the O moiety drives its preference for the I2M pathway.^18^ However, when the bda ligand is replaced with pda (Figure 1a, pda = 1,10-phenanthroline-2,9-dicarboxylate), it can only adopt the WNA pathway, resulting in a significant decrease in the catalytic activity to 0.102 s^–1^.^19^ Notably, there are no significant differences in the parameters that impact reaction pathways between WNA and I2M pathways, including the high spin nature of the Ru-oxo in these two Ru-type complexes.^20^ This change in reaction pathways is attributed to the incorrect orientation of the Ru-pda-type complex, i.e., hydrophobic oxo tends to point to a more hydrophobic phenanthroline moiety on another catalyst (Figure 1b), which leads to an additional rearrangement to form the favorable face-to-face configuration.^21^ Confining Ru-pda through covalent bonds within a polymeric film can switch the O–O bond formation pathway to I2M;^22,23^ however, the confinement in mesoporous silicas in a noncovalent manner does not significantly alter the catalytic mechanism (TOF = 0.133 s^–1^, TON = 1300, pH = 0.22).^24^ This implies that the microenvironment and supramolecular orientation between catalysts may significantly influence the O–O bond formation pathway. Note that loading catalysts with the WNA mechanism onto the electrode surface eliminates the need to consider possible switches in their catalytic mechanism, making them more convenient for practical applications.^25,26^ The studies on the Ru-bnda (bnda = 2,2′-bi(nicotinic acid)-6,6′-dicarboxylate) also indicated that the favorable intermolecular face-to-face interaction is an additional factor for the generation of the prereactive [Ru^V^=O···O = Ru^V^].^20^ In addition, Concepcion confirmed that entropy contributed significantly to the whole reaction barrier.^27^ A step forward, preorganization of the two metal-oxo species into the prereactive dimer was identified as the key step for the bimolecular O–O bond formation of Ru-bda catalysts by Ahlquist.^28^ This is why the strong π–π interaction of the axial ligand promotes the intermolecular O–O bond formation, leading to a 10-fold rate increase from [Ru(bda)pic_2_] (pic = picoline) to [Ru(bda)isoq_2_] (isoq = isoquinoline).^9^ Unfortunately, π–π interaction-induced dimerization is not a general strategy for mechanism tuning. For instance, introducing isoquinoline into a terpyridine catalytic system decreases its catalytic activity,^29^ emphasizing the importance of understanding the origins of these two different mechanisms.

Those previously insightful researches imply that both the entropy-driven geometric organization and enthalpy terms are necessary to be considered in the analysis of catalytic rates and mechanisms. To confirm this hypothesis, it would be intriguing to investigate whether the homogeneous Ru-pda catalyst could adopt an I2M mechanism through structural modifications that facilitate the coupling of high-valent Ru–oxo species.^30^ In this regard, we synergistically applied the high spin density of metal-oxo and preferable geometry preorganization to the Ru-pda-type catalyst (Figure 1b), which, as expected, promoted the intermolecular O–O bond formation pathway. Alongside a 70-fold increase in activity, theoretical studies provided a profound understanding of the I2M mechanism, underscoring its importance for advancing efficient catalytic systems.

## Results and Discussion

**Scheme 1:**
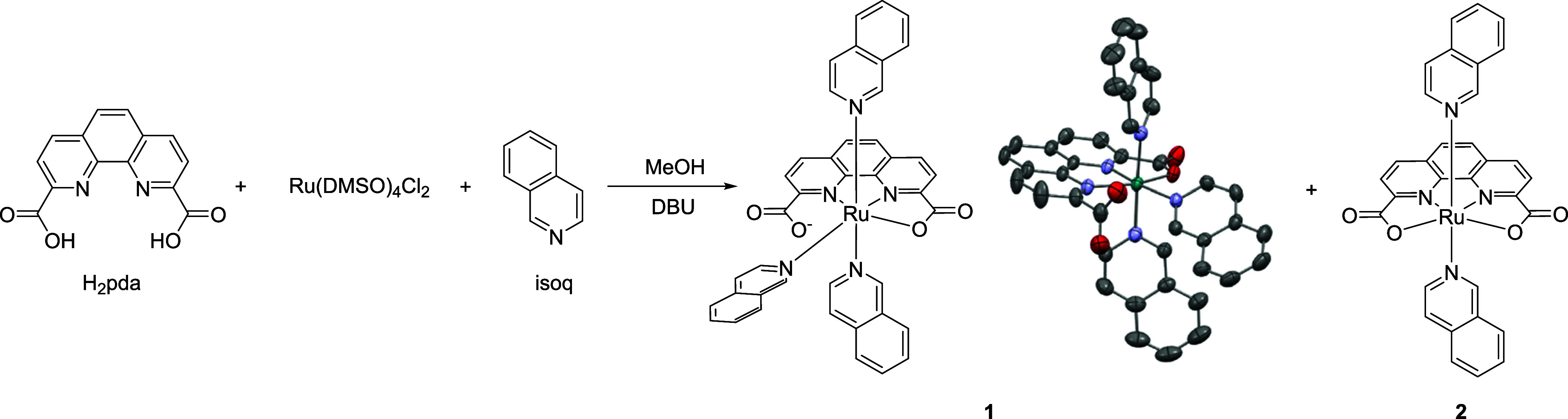
Synthetic Scheme of Complexes **1** and **2** and Crystal Structure of **1** with Thermal Ellipsoids at 50% Probability Hydrogen atoms are omitted for clarity.

The preparation of complexes **1** and **2** is detailed in the Experimental Section (Scheme 1). According to the ^1^H NMR spectra displayed in Figure S2, complex **2** demonstrates a symmetric structure, as indicated by three distinct signals corresponding to the pda unit (H_a_, H_b_, and H_c_). However, the addition of an extra isoquinoline at the equatorial position in complex **1** disrupts this symmetry, causing H_a_ to split into two separate peaks and broadening the H_b_ signal. Both six-coordinate complexes **1** and **2** exhibit typical diamagnetic properties, consistent with (***t***_**2g**_)^6^(***e***_**g**_)^0^ electronic configurations, suggesting that the metal centers are in the Ru^II^ oxidation state.

Complex **1** was crystallized by the slow diffusion of diethyl ether into its methanolic solution at room temperature. The resulting crystal structure revealed a slightly distorted octahedral geometry with Ru–N and Ru–O bond lengths of approximately 2 Å (Scheme 1 and Figure S1). This geometry is comparable to that of the [Ru^II^(bdaH)(isoq)_2_(NCCH_3_)](ClO_4_) analogue.^31^ In the structure, monodentate isoquinoline ligands are positioned both axially and equatorially. The equatorial pda ligand coordinates with the Ru center through three atoms (κ-N^2^O), leaving one carboxylate group uncoordinated. Due to the absence of an anionic counterion in the unit cell, this carboxylate group is likely deprotonated to balance the positive charge of the Ru^II^ center.

### Electrochemistry and Structural Evolution

The electrochemical properties of complex **1** were evaluated in aqueous solution (pH 1) with 10% CF_3_CH_2_OH by means of cyclic voltammograms (CVs) and differential pulse voltammograms (DPVs). Three oxidation peaks and three reduction peaks were observed (Figure S5a). Pourbaix diagram indicates that the corresponding three redox processes are 1e^–^ transfer, 2e^–^/1H^+^ transfer, and 1e^–^/1H^+^ transfer processes, respectively (Figure S5b). The first oxidation peak at *E*_Ox1_ = 0.85 V is much larger than the first reduction peak at *E*_Re1_= 0.78 V, suggesting that an adsorption phenomenon is likely involved in the oxidation process. The scan rate experiment showed that the peak current for the reduction process is controlled by diffusion, while the catalyst appears to adsorb onto the electrode during the initial oxidation step (Figure S6).^32^ This adsorption may be due to the interaction between the electrode and the equatorial isoquinoline ligand. As oxidation proceeds, the isoquinoline ligand may detach, resulting in the desorption of the catalyst from the electrode. Different active species are expected for the second oxidation (*E*_Ox2_ at 1.12 V) and reduction (E_Re2_ at 0.88 V) peaks due to the larger peak-to-peak separation. In addition, the second reduction wave appears only after reaching the *E*_Ox2_ (Figure S5a), suggesting that ligand exchanges occurred during the second oxidation process. The slope for the second redox process in the Pourbaix diagram is −27.9 mV/pH, indicating a 2-electron/1-proton transfer process where one electron is removed from the metal and the other from the isoquinoline ligand. The DPV of the isoquinoline ligand displayed two oxidation signals, with the position of the second peak overlapping somewhat with that of the Ru^IV/III^ peak (Figure S5c). This confirms that one of the electrons removed might be from the detached isoquinoline ligand. Taking the data together into consideration, we propose the structural evolution of complex **1** in Figure S5d.

### Water Oxidation by [Ru(pda)isoq_2_]

**Figure 2:**
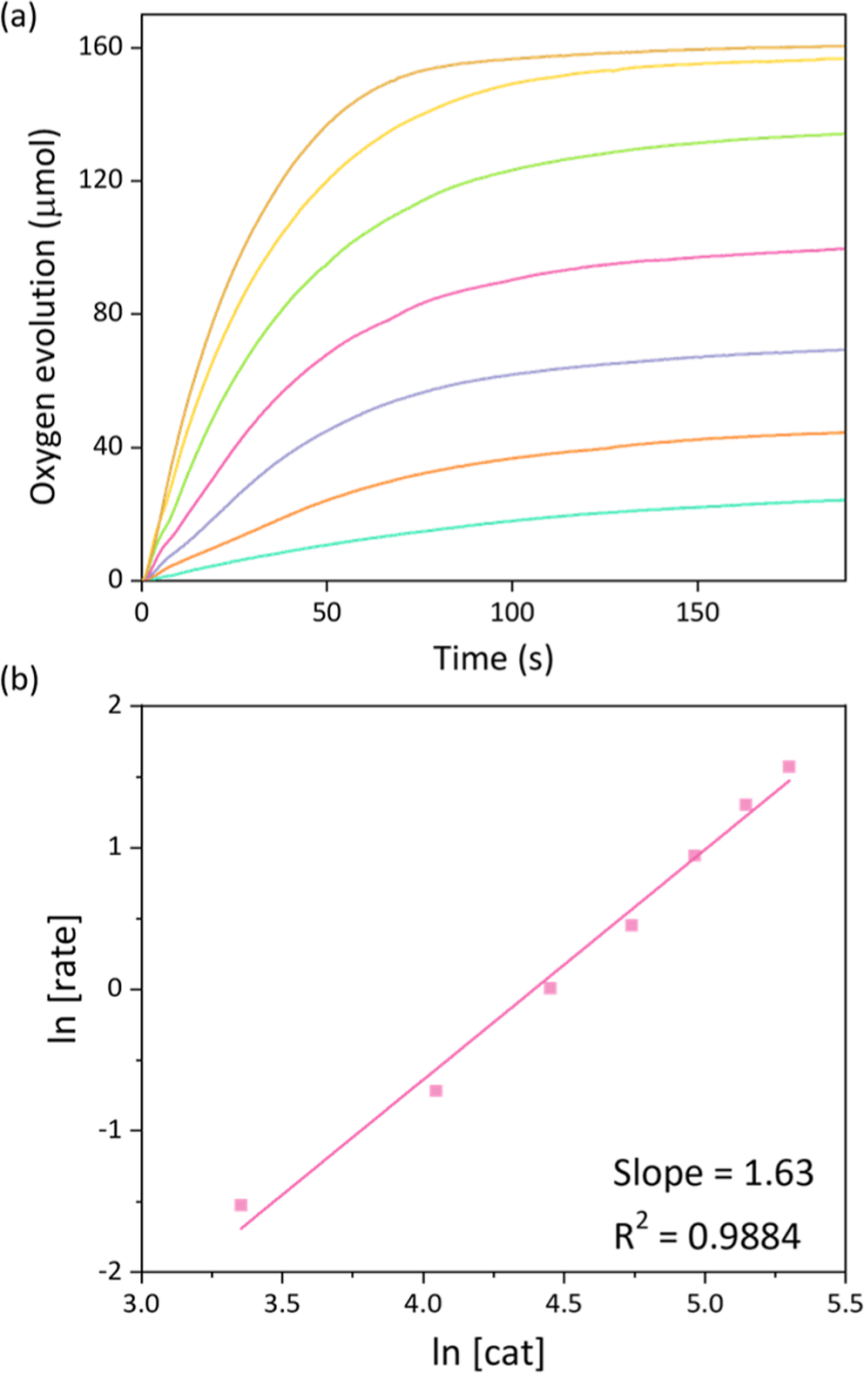
(a) Oxygen evolution vs time at various concentrations of catalyst **1** (30–200 μM) in pH 1 CF_3_COOH solution containing [Ce^IV^] = 0.17 M and (b) corresponding reaction order determinations.

The catalytic water oxidation activity of **1** was measured in pH 1 aqueous solution using ceric ammonium nitrate (CAN) as an oxidant. A previous study showed that the catalytic mechanism of [Ru(pda)pic_2_] was WNA;^11^ on the contrary, the reaction order between [Ru(pda)isoq_2_] and oxygen evolution rate was determined to be 1.63 as shown in Figure 2, reflecting that both mechanisms of WNA and I2M are involved. The secondary kinetic isotope effect (KIE) also confirmed that the breaking of the O–H-bond was not involved in the RDS, suggesting that the I2M was the dominant pathway (Figure S7). Thanks to this effective O–O bond formation pathway, the TOF values were accordingly increased by 2 orders of magnitude compared to that of [Ru(pda)pic_2_]^19^ (2–7 vs 0.04–0.1 s^–1^; Figure S8). In addition, the p*K*_a_ values for isoquinoline and pyridine are 5.14 and 5.23, respectively, indicating that the electronic changes of the axial ligands are unlikely to be responsible for the enhanced performance. A similar conclusion was confirmed for Ru-bda.^33,34^

The spin densities of oxyl species in Ru^V^(bda)pic_2_=O and Ru^V^(pda)pic_2_=O catalysts (Figure S9) with negatively charged equatorial ligands were found to be 0.722 and 0.711, respectively. These values are higher compared to the spin density of Ru^V^(bpc)(bpy)=O oxo (bpc = 2,2′-bipyridine-6-carboxylate), 0.604 from density functional theory (DFT) calculations. However, under homogeneous conditions, only Ru^V^(bda)pic_2_=O forms the O–O bond through intermolecular coupling, whereas both Ru^V^(bpc)(bpy)=O and Ru^V^(pda)pic_2_=O catalysts react via the WNA pathway. These data collectively indicate that high spin density of metal-oxo alone does not imply the I2M mechanism. To investigate the preorganization effect on reaction pathways, the Ru(pda)(isoq)_2_ model was parameterized (see the Supporting Information for details) and filled with the TIP3P water model for empirical valence bond (EVB) and molecular dynamics (MD) simulations. The hydrogen bond analysis shows that the oxo of [Ru^V^(pda)isoq_2_=O] forms only 0.01 hydrogen bonds with water molecules, while the oxygen atoms of the carboxylate group form 0.12 and 0.34 hydrogen bonds (Figure S11). This indicates that the oxo is hydrophobic, which could be due to its low charge (−0.152) compared to the carboxylate oxygen atoms (−0.454 and −0.484) (Table S3). The radial distribution function analysis of the oxo atom with H_2_O molecules (Figure S13) also proves the hydrophobic oxo, with the first solvation shell of the oxo being approximately 3.2 Å. The result is consistent with the observations in [Ru^V^(bda)=O] and [Ru^V^(pda)pic_2_=O] species, as confirmed by DFT calculations with several surrounding water molecules and EVB-MD simulations.^18,21^ However, the hydrophobic oxo in [Ru^V^(pda)pic_2_=O] favors a specific geometry where one oxo points toward the hydrophobic pda backbone of another species (front-to-back configuration).^21^

**Figure 3:**
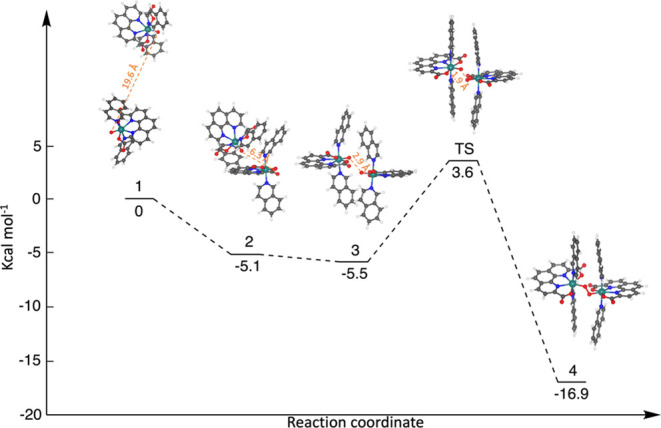
Free-energy profile for the formation of two [Ru(pda)isoq_2_] catalysts via the I2M reaction pathway in the water phase. The snapshots presented in the profile are a configuration of two catalysts positioned relatively far apart (1), a front-to-back configuration (2), a prereactive dimer (3), the transition state (TS), and a product (4), which were obtained from umbrella sampling and EVB-MD simulations. The TIP3P water molecules have been omitted to enhance clarity.

To explore the energy variation in different arrangements of two [Ru^V^(pda)isoq_2_=O] species, umbrella sampling simulations were conducted for the movement of one species from the prereactive dimer configuration (**3** in Figure 3) toward the first solvation sphere of another species, subsequently extending to a relatively distant position (**1** in Figure 3). Throughout the three repeated simulations, one key configuration, namely, the front-to-back configuration (**2** in Figure 3), which is observed in 100 ns MD simulations, displayed a mean binding free energy of approximately 5.1 kcal mol^–1^. In this front-to-back configuration, one of the isoquinoline ligands in two species aligns parallel to each other, while an oxo group points toward the pda backbone of another species. However, the prereactive dimer geometry (**3** in Figure 3) where the two isoquinoline ligands of two species form two parallel π-stacking interactions is approximately −0.4 kcal mol^–1^ more favorable in binding free energy compared to the front-to-back configuration. Comparatively, the [Ru^V^(pda)pic_2_=O] complex requires a rearrangement-free energy of 3.0 kcal mol^–1^ to rearrange from the front-to-back geometry to the correct prereactive complex. Hence, the formation of two π-stacking between the isoquinoline ligands of the two species in the [Ru^V^(pda)isoq_2_=O] complex would be one of the driving forces to form the prereactive dimer configuration, similar to the [Ru^V^(bda)isoq_2_=O] complex. Once the prereactive dimer configuration is established, the O–O bond formation step in the Ru(pda)isoq_2_ complex is energetically favorable with a reaction free energy of −12.2 kcal mol^–1^ and an activation free energy of 6.3 kcal mol^–1^ in an implicit solvent model (Table S2). The reaction free energy and activation free energy will shift to −11.4 and 9.1 kcal mol^–1^, respectively, under the EVB-MD simulations of the O–O coupling reaction in the TIP3P water model (Figure 3). Conversely, the WNA pathway is endergonic with a reaction energy of 13.2 kcal mol^–1^ and an activation free energy of 22.4 kcal mol^–1^ (Figure S16). Therefore, the formation of the prereactive dimer was facilitated by the parallel π-stacking interactions. This dimer undergoes the I2M pathway with a low activation free energy, leading to a faster reaction rate for [Ru^V^(pda)isoq_2_=O] compared to that for [Ru^V^(pda)pic_2_=O] (Figure S17).

## Conclusions

In summary, the intermolecular directionality of the catalyst can be synergistically tuned by negotiation of the spin density on oxo and the preferable organizational geometry of the catalysts. Our previous theoretical calculations showed that the hydrophobic/hydrophilic directionality was responsible for the mechanism switch between the Ru^V^(bda)pic_2_=O and Ru^V^(pda)pic_2_=O (from I2M to WNA). In this work, we experimentally demonstrate the impact of hydrophobic/hydrophilic directionality on the O–O bond formation mechanism by modifying the Ru-pda-type catalyst with isoquinoline axial ligands. By the radical coupling O–O bond formation pathway, a rate increase of 2 orders of magnitude was achieved for Ru^V^(O)(pda)isoq_2_ compared to its structural analogue Ru^V^(O)(pda)pic_2_. Structure analysis found that the isoquinoline complex has a large probability of forming the prereactive dimer structure due to the formation of π–π stacking among the isoquinoline ligands. Calculations on the activation free energy show that the I2M pathway of Ru^V^(O)(pda)isoq_2_ complex has a lower activation free energy, leading to a higher reaction rate in Ru-pda-type catalysts.

## Experimental Section

### Synthesis and Characterization

A mixture of 1,10-phenanthroline-2,9-dicarboxylic acid (H_2_pda, 266 mg, 1 mmol), [Ru(DMSO)_4_Cl_2_] (484 mg, 1 mmol), and 600 mg of 1,8-diazabicyclo[5.4.0]undec-7-ene (DBU) in methanol (5 mL) was heated at 100 °C for 3 min under microwave irradiation (Scheme 1). Then, an excess amount of isoquinoline (774 mg, 6 mmol) was added and heated under microwave irradiation at 100 °C for 1 h. The crude product was purified by column chromatography (Al_2_O_3_, dichloromethane: methanol 50:1) to afford 75 mg of **1** (yield: 10%) and 7 mg of **2** (yield: 1%). ^1^H NMR of complex **1** (500 MHz, MeOD) δ 9.39 (s, 1H), 8.95 (s, 2H), 8.57 (d, *J* = 8.2 Hz, 1H), 8.46 (d, *J* = 8.2 Hz, 1H), 8.35 (d, *J* = 8.0 Hz, 2H), 8.14 (s, 2H), 7.93–7.85 (m, 4H), 7.74 (dt, *J* = 18.6, 6.9 Hz, 6H), 7.66–7.60 (m, 4H), 7.56 (t, *J* = 7.4 Hz, 1H), 7.50–7.43 (m, 4H). ^13^C NMR (126 MHz, MeOD) δ 176.12, 171.29, 160.61, 159.88, 156.06, 153.22, 151.28, 150.84, 148.10, 145.99, 142.76, 138.69, 136.32, 133.43, 133.09, 132.86, 131.32, 130.74, 130.57, 130.21, 129.68, 129.24, 129.10, 128.52, 127.83, 127.67, 127.50, 127.43, 126.26, 125.52, 123.09, 122.67. HRMS: found *m*/*z*^+^ = 627.0619 (M - isoquinoline + H^+^), calcd for: 627.0615. ^1^H NMR of complex **2** (500 MHz, MeOD) δ 8.94 (s, 2H), 8.45 (d, *J* = 8.4 Hz, 2H), 8.35 (d, *J* = 8.4 Hz, 2H), 8.18 (s, 2H), 7.89–7.79 (m, 6H), 7.74 (t, *J* = 7.6 Hz, 2H), 7.63 (t, *J* = 7.6 Hz, 2H), 7.54 (d, *J* = 6.5 Hz, 2H). The equatorial isoquinoline ligand of **1** is labile, which is prone to be detached under ionization (Figure S5) and applied potential (Figure S6). Therefore, the same active species for complexes **1** and **2** are envisaged once reaching Ru^V^ states.

The majority of the byproducts are identified as Ru(pda)_2_. No uncommon hazards are noted with the experimental work.

## Supporting Information Available

General procedures, X-ray crystallographic data, NMR and HRMS spectra, electrochemistry data, and computational details (PDF)
