# Supplementary material for: Intermolecular O–O Bond Formation between High-Valent Ru–oxo Species
Source: Inorg Chem. 2024 Aug 19;63(35):16161–6. doi: 10.1021/acs.inorgchem.4c01560 (PMC11372747; doi:10.1021/acs.inorgchem.4c01560)
Supplement: Supplementary file 1 — ic4c01560_si_001.pdf [file ic4c01560_si_001.pdf]

Intermolecular O-O bond formation between high valent Ru–oxo species

Tianqi Liu[1,2‡], Shaoqi Zhan[3,4‡], Biaobiao Zhang[5‡], Linqin Wang[5], Nannan Shen[6], Mårten S. G. Ahlquist[1], Xiaolei Fan[2, 7] and Licheng Sun[1,5*]

[1] Department of Chemistry, School of Engineering Sciences in Chemistry Biotechnology and Health, KTH Royal Institute of Technology, 10044 Stockholm, Sweden

[2] Institute of Wenzhou, Zhejiang University, Wenzhou 325006, China

[3] Department of Chemistry-BMC, Uppsala University, BMC Box 576, S-751 23 Uppsala, Sweden

[4] Department of Chemistry - Ångström Laboratory; Uppsala University, Box 523, 75120 Uppsala, Sweden.

[5] Center of Artificial Photosynthesis for Solar Fuels and Department of Chemistry, School of Science, Westlake University, 310024 Hangzhou, China

[6] State Key Laboratory of Radiation Medicine and Protection, School for Radiological and Interdisciplinary Sciences (RAD-X) and Collaborative Innovation Center of Radiation Medicine of Jiangsu Higher Education Institutions, Soochow University, 215123 Suzhou, China

[7] Department of Chemical Engineering, School of Engineering, The University of Manchester, Oxford Road, Manchester M13 9PL, United Kingdom

* Corresponding author: lichengs@kth.se

TL, BZ and LS conceived the project. TL performed the synthesis and electrochemical characterization and wrote the manuscript. SZ and MA were responsible for the calculation work. BZ performed the Ce^{IV}-driven kinetics measurement. TL, LW and NS grew and refined the crystal structure. TL, SZ and BZ contributed equally to this work.

General Procedures

All chemicals were purchased from commercial suppliers and used as received. All the solvents were purchased from Fisher Scientific Sweden and used as received. High resolution mass spectrometry was performed at the Dalian University of Technology and Westlake University (UPLC-HRTOF, Synapt-G2-Si, Waters). ^{1}H NMR and ^{13}C NMR spectra of the compounds were recorded with either a Bruker Ascend 400 or a Bruker Avance DMX 500 NMR spectrometer. Electrochemistry measurements were carried out with a CHI660c potentiostat, with glassy carbon as working electrode, Pt wire as auxiliary electrode and measured versus Ag/AgCl/Sat. KCl as a reference electrode. All potentials reported herein are converted to their corresponding values versus NHE. Kinetics studies were carried out with an Omega PXM409 pressure transducer.

Single-crystal growth and measurement. Single crystal of **1** was obtained by slow diffusion of diethyl ether into a methanolic solution of **1** at room temperature. The diffraction data of **1** was measured at 293 K by using Mo Kα radiation on a Bruker D8 VENTURE single crystal X-ray diffractometer equipped with a kappa geometry goniometer. The dataset was reduced, and absorption correction was applied in APEX3 suite. The crystal structure was solved and refined by SHELXL-2018 and OLEX2, respectively. (G. M. Sheldrick, Acta Crystallogr. Sect. A: Found. Crystallogr., 2007, 64, 112-122) The crystal structure was refined using full-matrix least-squares based on F^2 with all non-hydrogen atoms anisotropically defined. Hydrogen atoms were placed in calculated positions by means of the "riding" model. A summary of the crystallographic data, the data collection parameters, and the refinement parameters are given in Table S1.

Kinetic isotope effect (KIE) values calculation from electrochemical experiments

$$pK_{D_2O} = pOD + pD = 14.87$$

$$pK_{H_2O} = pOH + pH = 14$$

The CVS were recorded with a scan rate of 20 mV s^{-1} in 0.1 M phosphate buffer solution:

$pH_{reading}$ = 7.47, *i.e.* PD = 7.87 in D_2O (J. Phys. Chem. B 2019, 123, 8195–8202)

$pH_{reading}$ = 7 in H_2O

The overpotential can be expressed by the equations S1 and S2 in H_2O and D_2O.

$$\eta^{H_2O} = E_{RHE} - 1.229V_{RHE} = E^{reading}_{Ag/AgCl} + 0.059pH + E^{H}_{Ag/AgCl} - 1.229V_{RHE}(S1)$$

$$\eta^{D_2O} = E_{RHE} - 1.262V_{RDE} = E^{reading}_{Ag/AgCl} + 0.059pD + E^{D}_{Ag/AgCl} - 1.262V_{RDE}(S2)$$

The difference between $E^{H}_{Ag/AgCl}$ and $E^{D}_{Ag/AgCl}$ is −0.013 V. (Bard, A. Standard potentials in aqueous solution, (Routledge, 2017).)

Therefore, in our case,

$$\eta^{H_2O} - \eta^{D_2O} = 0.059 \text{ x } (7 - 7.87) + 0.033 = 0.00533 \text{ V (S3)}$$

which is negligible. Therefore, we reported the KIE using Ag/AgCl as the reference to maintain the same driving force.

Computational details. All density functional theory (DFT) calculations for estimating Gibbs free energies were performed using the Jaguar 8.3 program package from Schrödinger LLC.[1] We employed Becke's three-parameter hybrid functional combined with the LYP correlation functional (B3LYP), [2] along with D3 dispersion corrections as proposed by Grimme et al. [3, 4] The LACVP** core potential and basis set were used for the optimization of molecular geometries.[5] To identify the transition states for O–O bond formation, we scanned the potential energy surface by varying the terminal O–O bond distance in the [Ru^{V}═O···O═Ru^{V}] antiferromagnetic open-shell singlet state. The thermochemical corrections required to estimate the Gibbs free energy barrier from the prereactive dimers were computed at the B3LYP-D3/LACVP** level for both the prereactive dimers and the transition-state structures. Single-point energy corrections were applied using the B3LYP-D3 functional with the LACV3P**++ basis set, which includes additional f-functions on the metal. Based on these gas-phase optimized geometries, implicit solvation energies were estimated through single-point calculations employing the Poisson–Boltzmann reactive field (PBF) method in Jaguar, simulating water as the solvent. The Gibbs-free energy was defined by the following equation: G = E(B3LYP-D3/LACV3P**++ 2f on Ru) + G_{solv} + ZPE + H_{298} – TS_{298} + 1.9 kcal mol^{-1} (The value 1.9 kcal mol^{-1} is a concentration correction to the free energy of solvation, which by default is calculated at 1 M (g) to 1 M (aq) in Jaguar).

Force-Field Parameterization.

The geometries of both the reactant monomer and the product dimer were optimized using the DFT method outlined above. For the force field parameters of the Ru(pda)(isoq) complex, we

utilized data from previous research[6], supplemented with standard OPLS-AA (all-atom optimized molecular potential for liquid simulation)[7] bonded and van der Waals parameters for the isoquinoline ligand. Partial charges were determined using ESP charges, following the methodology described in earlier work. [6] These parameters were then converted into the GROMACS topology format. The Fourier coefficients of the dihedral potential term were transformed into the Ryckaert–Bellemans form to facilitate molecular dynamics (MD) simulations. [8]

MD Simulations. MD simulations were performed with the GROMACS 5.0.4 MD software package.[9] We performed a 100 ns MD run in a 52 × 50 × 50 $Å^3$ periodic box filled with TIP3P[10] water molecules and a chloride ion to neutralize the charge. In MD simulations, the resulting systems were subject to 100,000 steps of steepest descent minimization. The periodic boundary condition was applied in the simulation. The cutoff radii for the Lennard-Jones and electrostatic interactions were set to 10 Å. For accurate evaluation of long-range Coulombic interactions, the particle mesh Ewald[11] summation method is used for electrostatic interactions beyond the cutoff. The system was gradually heated to 300 K over 100 ps using a v-rescale thermostat[12] for canonical ensemble (NVT) simulations. During this phase, the linear constraint solver algorithm[13] was utilized to constrain all bond lengths. Subsequently, isothermal-isobaric ensemble (NPT) simulations were performed, maintaining the pressure at 1 bar over 100 ps, controlled by a v-rescale thermostat and the Parrinello-Rahman barostat. The systems were then simulated for an additional 100 ns. Three independent MD simulations with different initial velocities were also carried out. A time step of 2.0 fs was used for all simulations. Trajectories were recorded every 200 ps (100,000 timesteps), and the final 50 ns of each trajectory were used to calculate the mean square displacement and obtain the diffusion coefficient of the catalyst.

Potential of Mean Force. The PMF calculations were performed for the two species of Ru(pda)(isoq) in the water phase using the umbrella sampling method with the GROMACS 5.0.4 MD software package. Equilibrations were performed for 100 ps under an NVT ensemble, using the same methodology described above. Two Ru complexes were pulled apart using a spring constant of 30 kJ mol^{-1} $Å^{-2}$ and a pull rate of 0.05 Å ps^{-1}. From these trajectories, snapshots were taken to generate the starting configurations for the umbrella sampling windows. Then, we sampled the distances using roughly 0.5 Å spacing. Such spacing allowed for increasing details at

a smaller distance and resulted in windows. In each window, a 100 ps isothermal–isobaric ensemble (NPT) simulation, with the pressure set to 1 bar in, was used. Followed by a 10 ns MD run for a total simulation time utilized for umbrella sampling. Analysis of results was performed with the weighted histogram analysis method.[14] All PMF simulations resulted in smooth dissociation curves, and the mean value was obtained from three repeated simulations.

EVB Simulations. The EVB simulations were conducted in both vacuum and aqueous phases to investigate the influence of the interface on the dimerization of the Ru(pda)(isoq) complex. Each molecular dynamics (MD) simulation involved sequential equilibration of the catalyst under conditions consistent with prior studies. The EVB simulations and mapping procedures followed the strategy detailed in previous work.[15]

Table S1 Summary of the crystallographic data for **1** (CCDC 2133354)

	**1**
Empirical formula	$C_{41}H_{27}N_5O_4Ru$
Formula weight	754.77
Crystal system	monoclinic
Space group	P 2/c
a/Å	18.7130(15)
b/Å	12.5294(9)
c/Å	14.8081(11)
α/°	90
β/°	92.785(3)
γ/°	90
V/Å^3	3467.8(5)
Z	4
T/K	293
λ/Å	0.71073
F(000)	1536
ρ_{calcd}/g cm^{-3}	1.446
μ/mm^{-1}	0,502
Measured refls.	7701
Independent refls.	6510
No. of parameters	460
R_{int}	0.0381
R_1 (I> 2σ(I))[a]	0.0279
ωR(F^2) (I> 2σ(I))[b]	0.0759
GOF	1.053

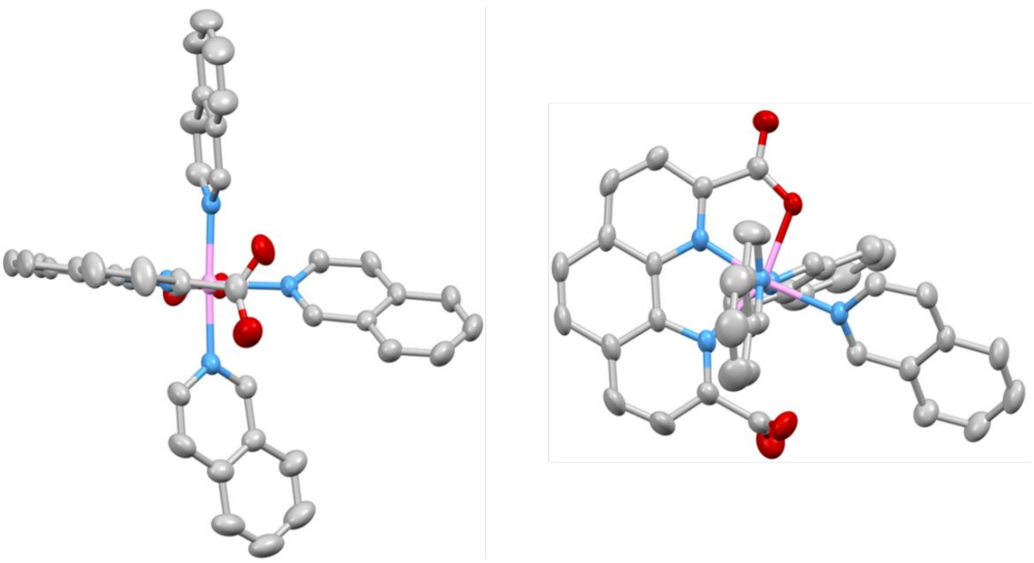

Figure S1. Single crystal structures of **1** with thermal ellipsoids at 50% probability. Hydrogen atoms are omitted for clarity.

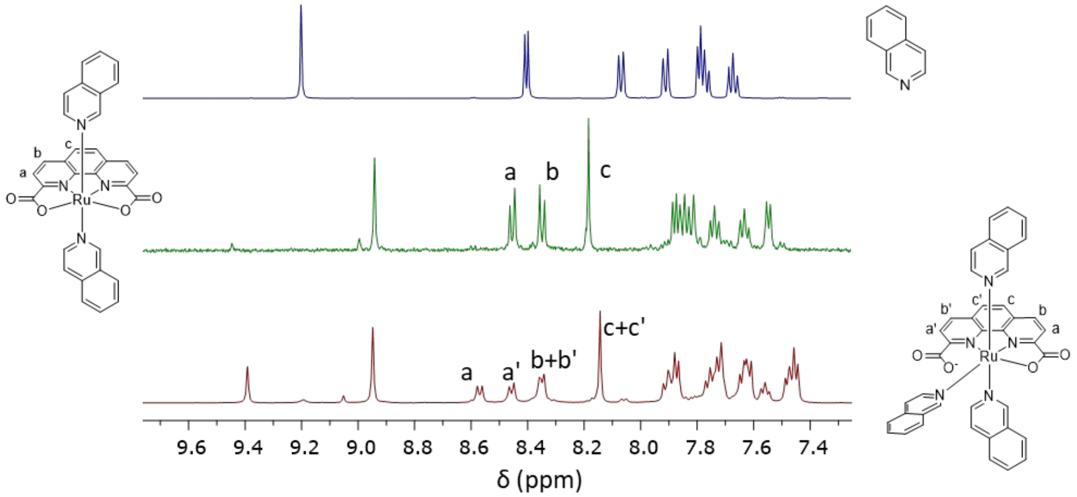

Figure S2. ^{1}H NMR spectra of complex **1**, **2** and isoquinoline in CD_3OD.

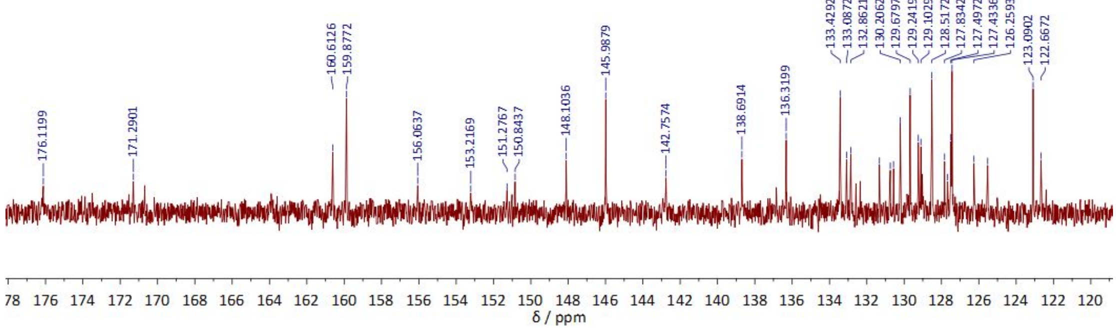

Figure S3. ^{13}C NMR spectra of complex **1** in CD_3OD.

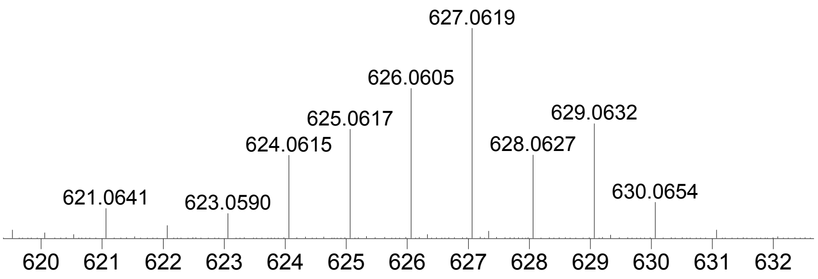

Figure S4. HRMS of complex **1**.

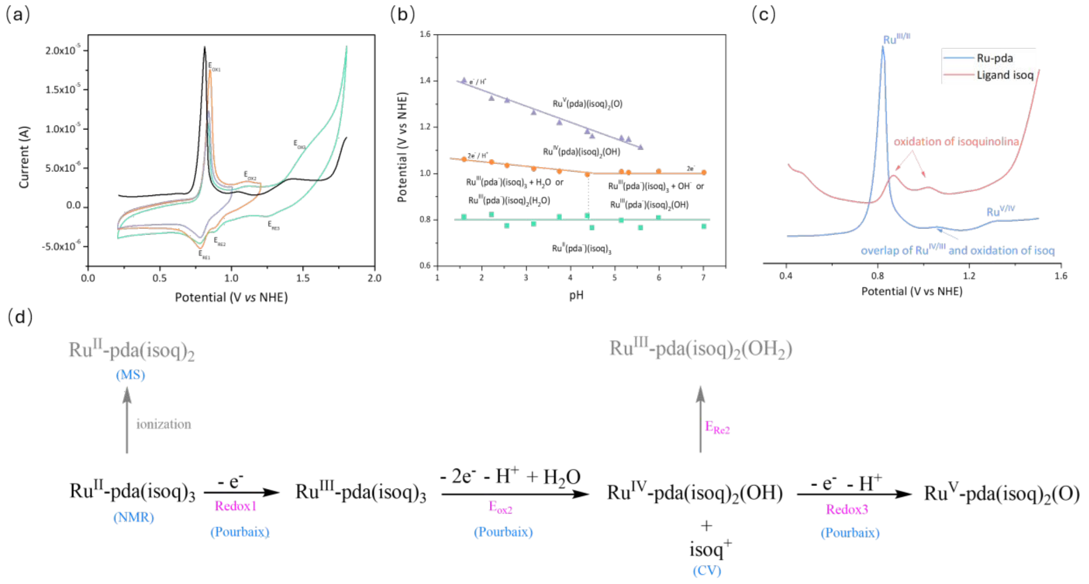

Figure S5. (a) CVs and DPV of complex **1** in pH 1 triflic acid solution containing 10% CF_3CH_2OH, scan rate = 100 mV s^{-1}, working electrode: GC. (b) Pourbaix diagram of complex **1**. (c) DPVs of complex **1** and isoquinoline ligand in pH 1 triflic acid solution containing 10% CF_3CH_2OH. (d) Proposed structural evolution of the catalyst.

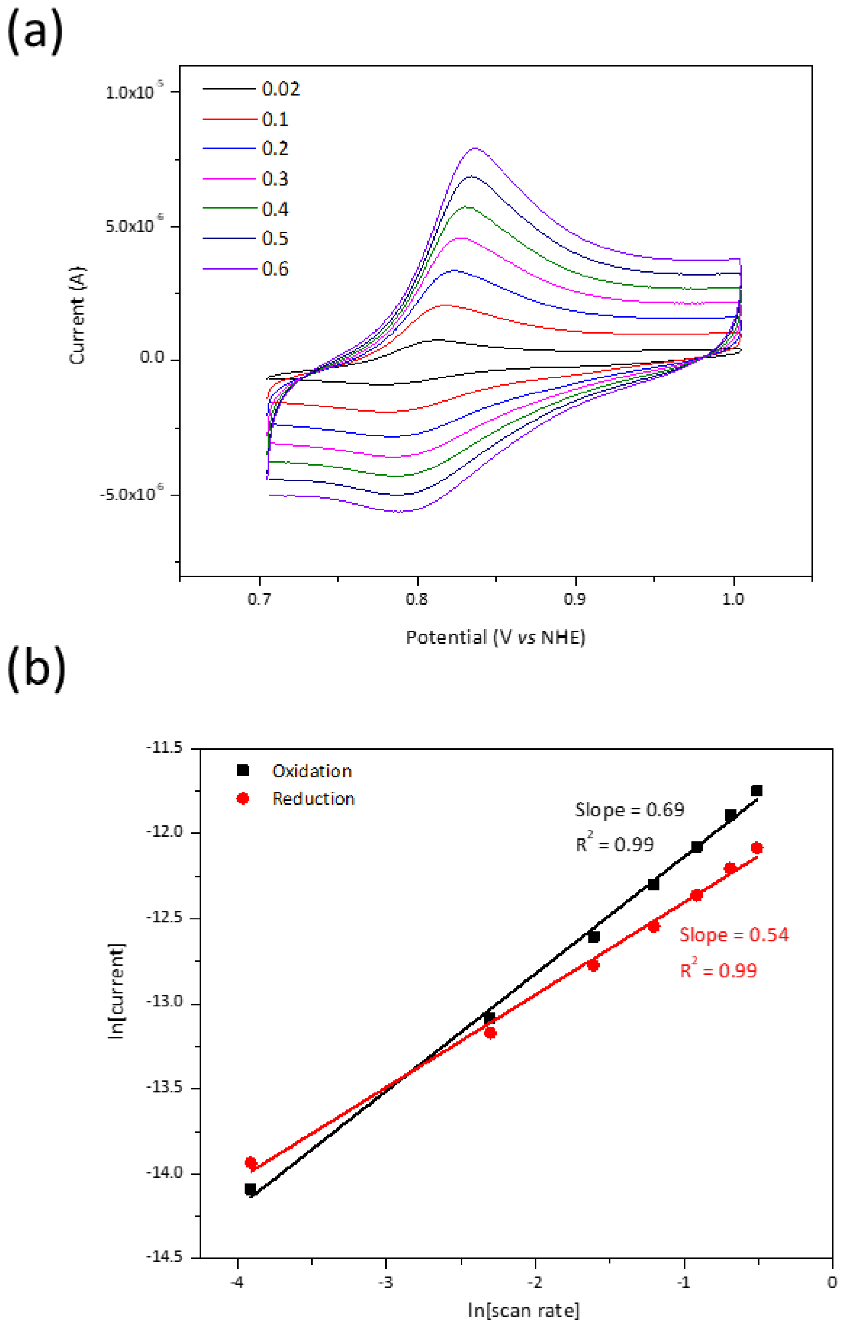

Figure S6 (a) CVs of complex **1** at different scan rates from 0.02 to 1 V s^{-1}, working electrode: GC. (b) Reaction order between peak current (E_{OX1} and E_{RE1}) and the scan rate. The slope of 1 indicates that this process is surface-controlled, while the slope of 0.5 indicates that this process is diffusion-controlled.

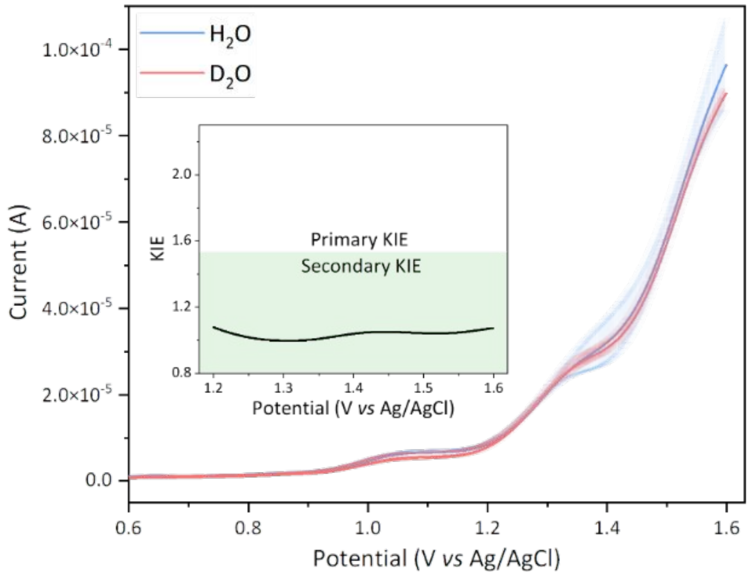

Figure S7 LSVs of 0.7 mM complex **1** in a 0.1 M phosphate buffer solution in H_2O and D_2O, (pH = 7 and pD = 7.87) containing 10% CF_3CH_2OH, scan rate = 100 mV s^{-1}, working electrode: GC. Each curve is the mean of the data fitting of three replicate experiments, and the shaded areas show the standard deviations for each series.

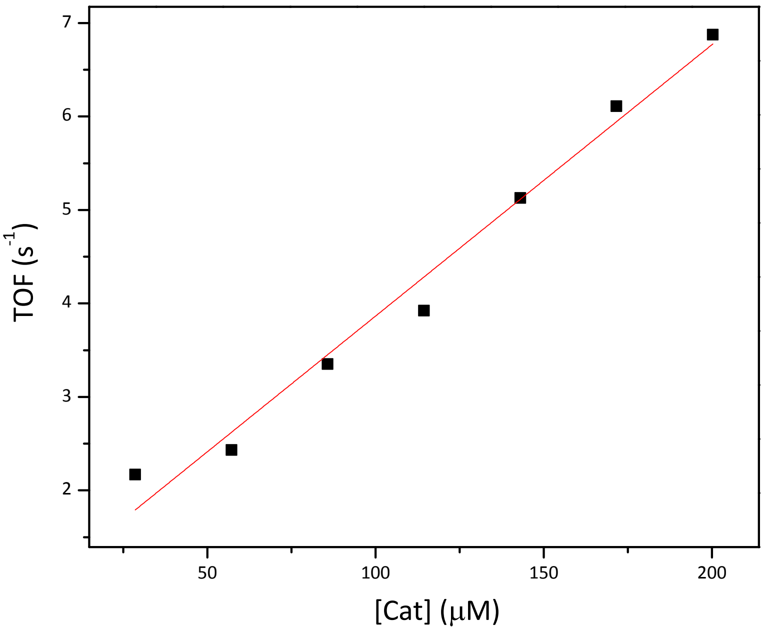

Figure S8 TOFs of complex **1** at various concentrations.

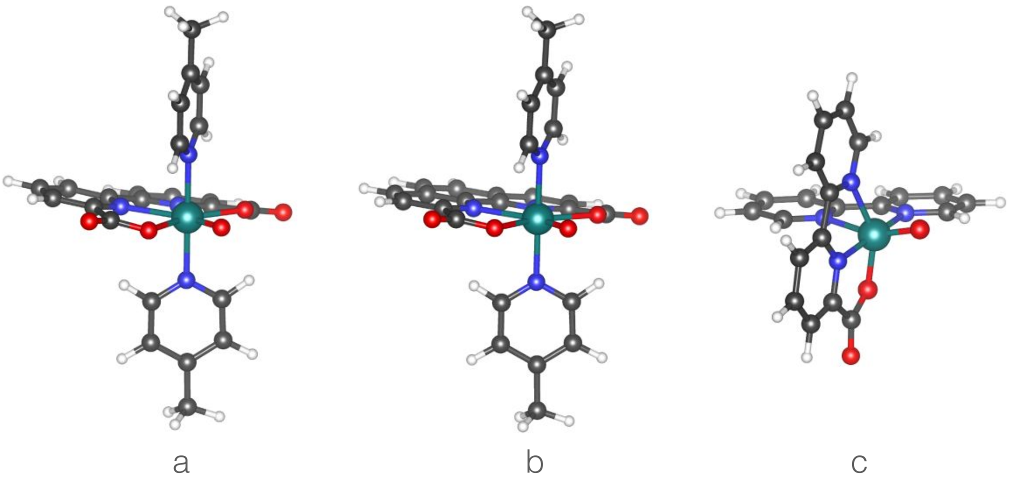

Figure S9 DFT optimized geometries of the Ru(bda)pic$_2$ (a), Ru(pda)pic$_2$ (b), and Ru(bpc)(bpy) (c).

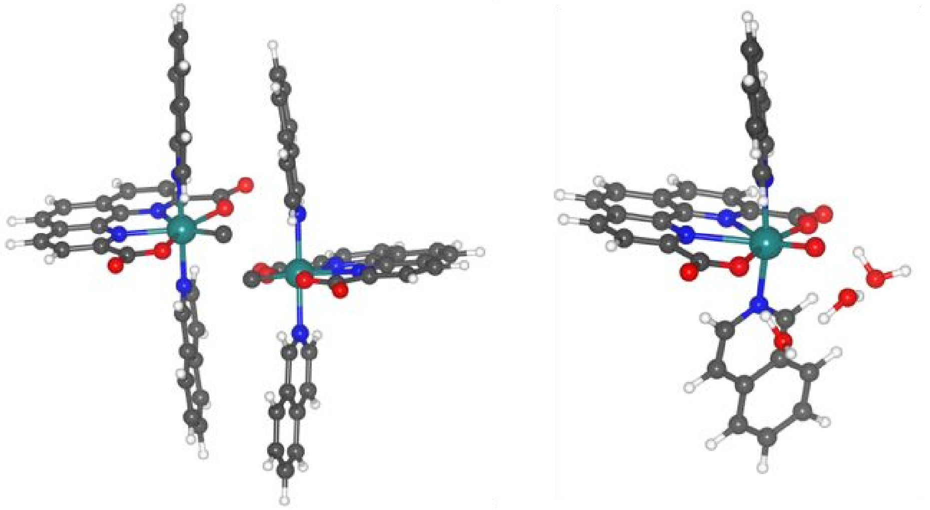

Figure S10 DFT optimized geometries of the transition state in the I2M (left) and WNA (right) reaction pathway for Ru(pda)isoq$_2$.

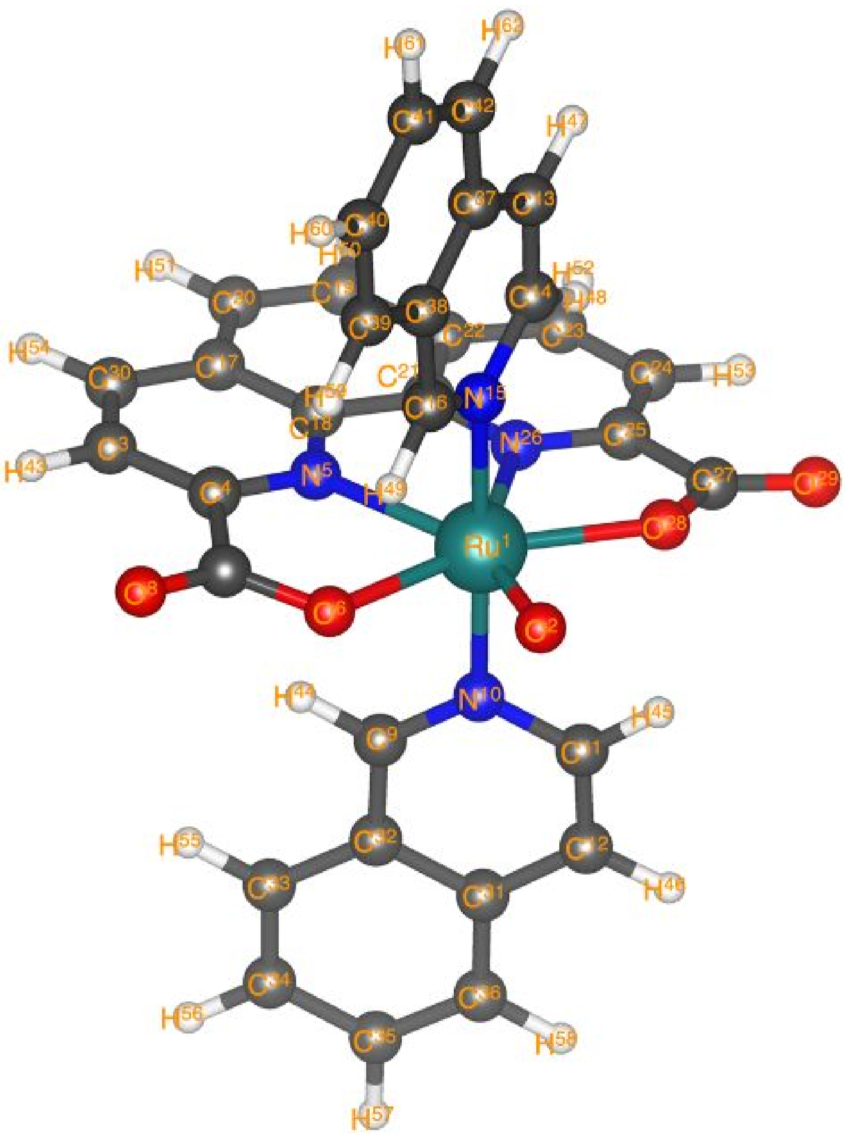

Figure S11 DFT optimized geometries of the Ru(pda)isoq$_2$ with labelled atoms.

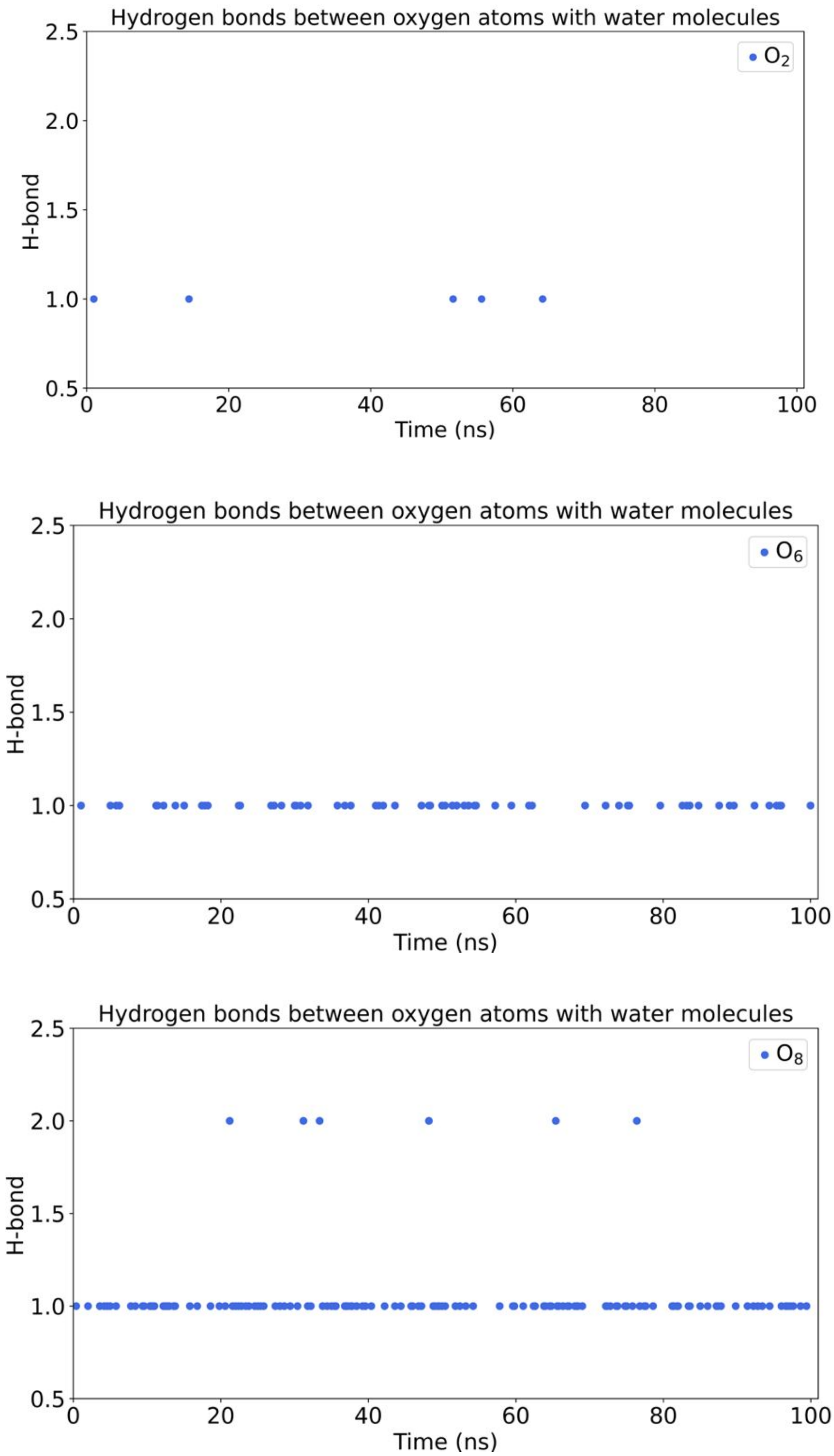

Figure S12 Hydrogen bonds formed between oxygen atoms and water molecules from MD simulations.

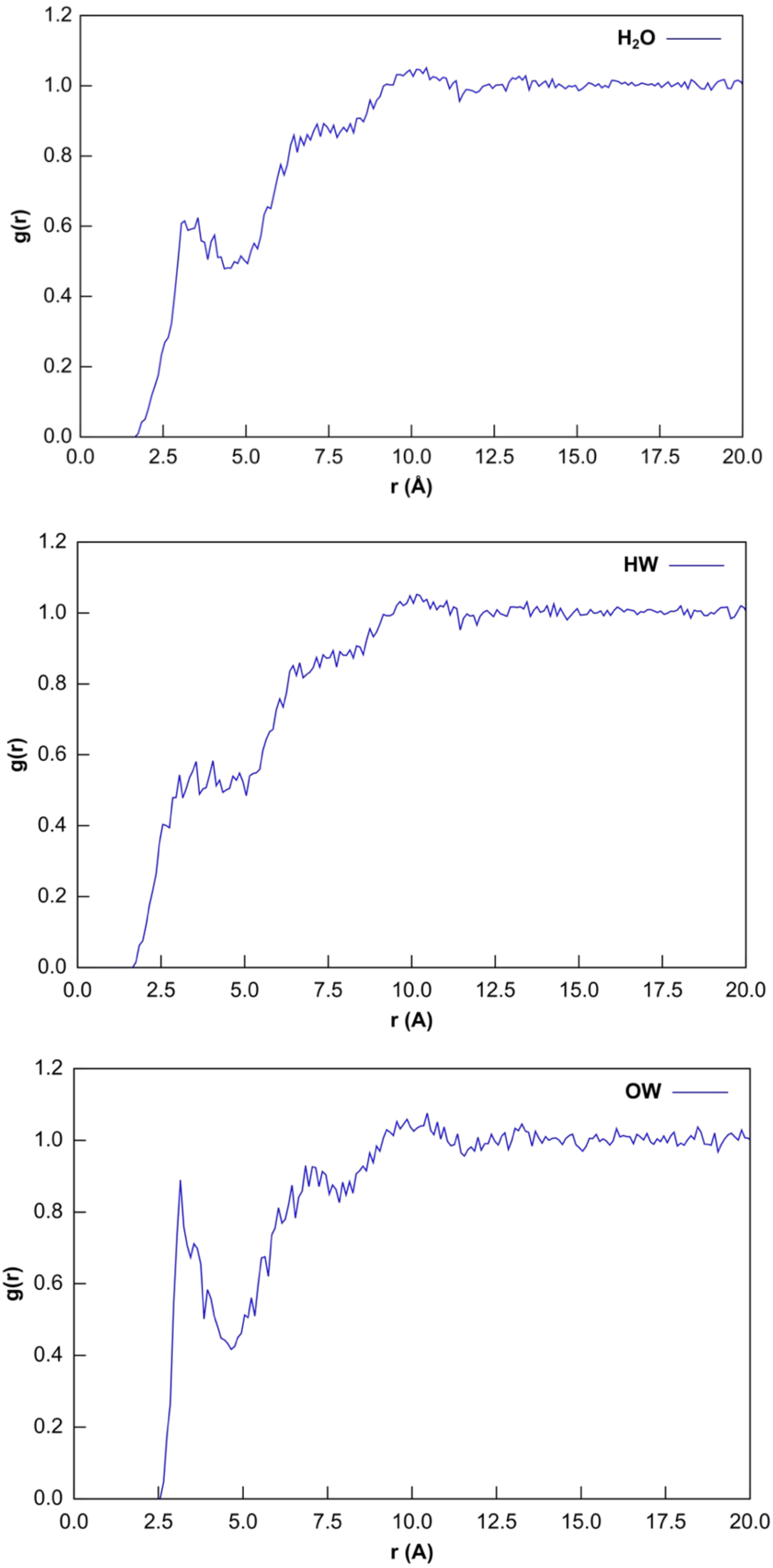

Figure S13 Radial distribution function between the oxo atom and water molecules from MD simulations.

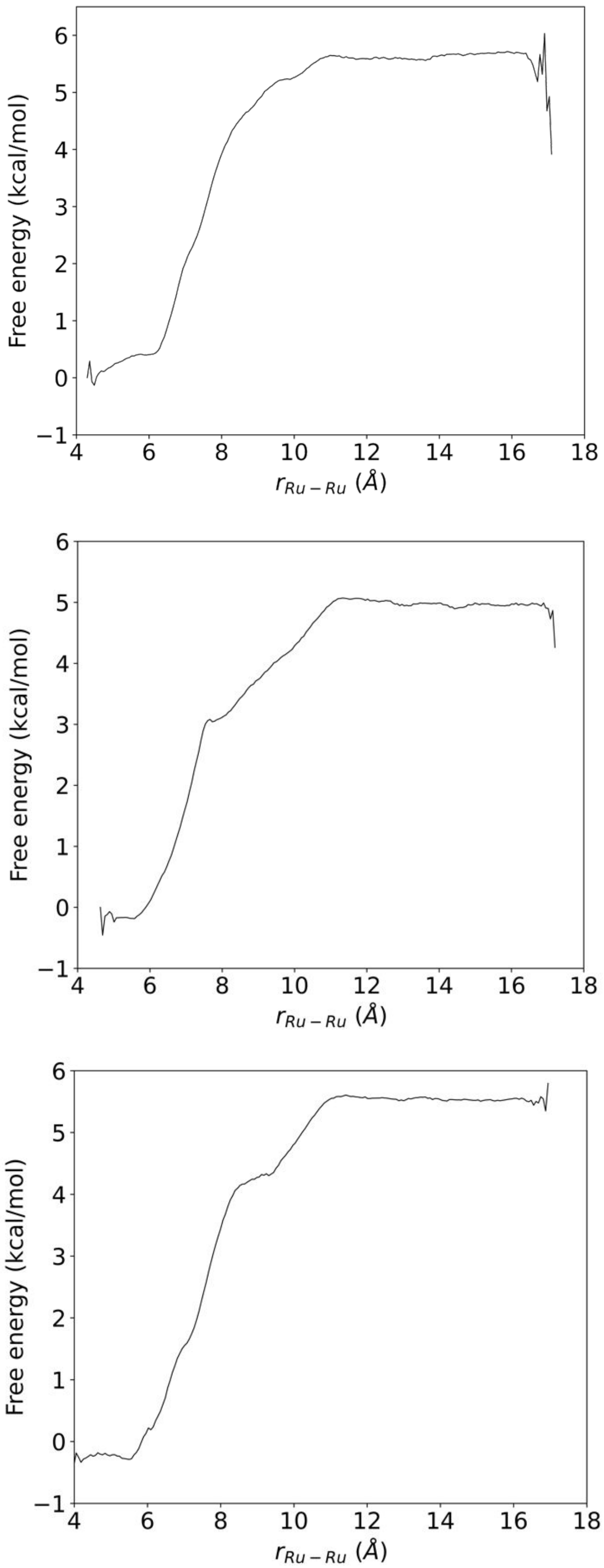

Figure S14 Free energy profile for the binding of two [Ru(pda)isoq$_2$] catalysts in the water phase from three replicated umbrella sampling simulations. The reaction coordinate corresponds to the distance between the Ru atoms of the two [Ru(pda)isoq$_2$] catalysts.

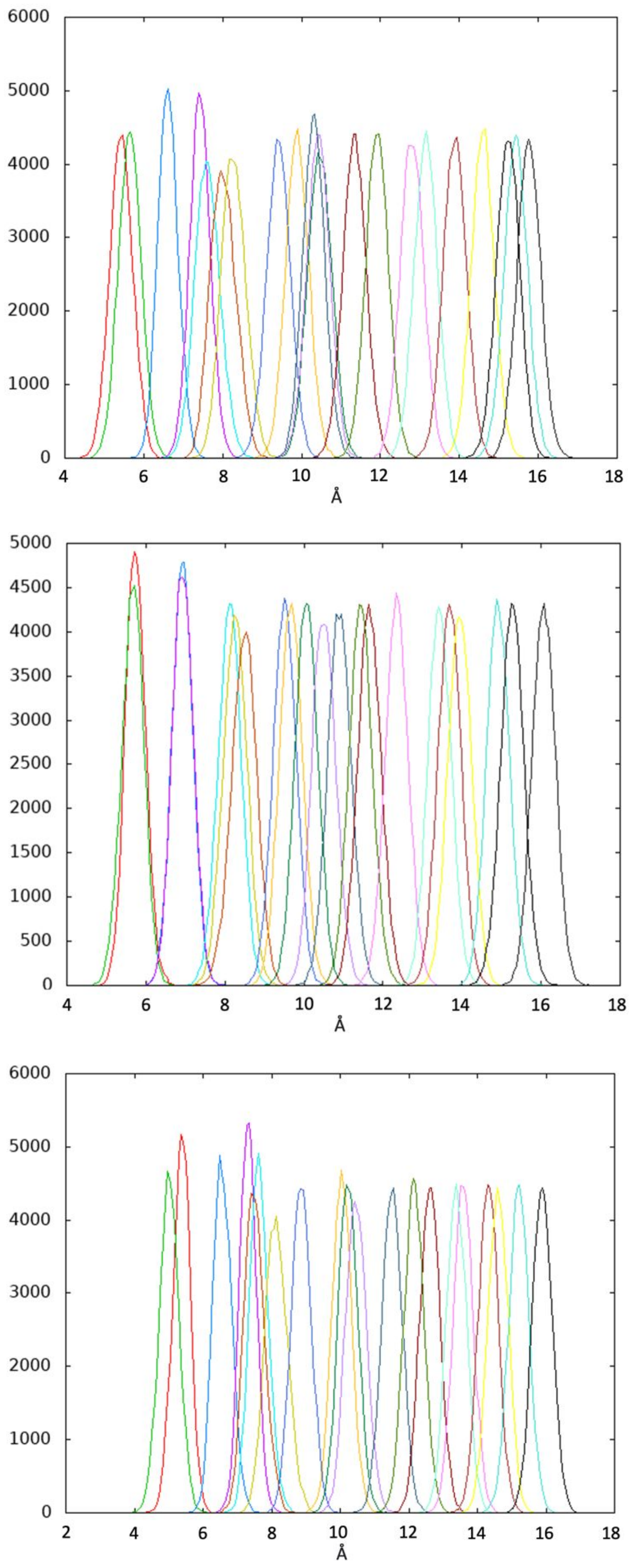

Figure S15 Histogram of the reaction coordinates from three replicated umbrella sampling simulations for calculating binding free energy with the WHAM.

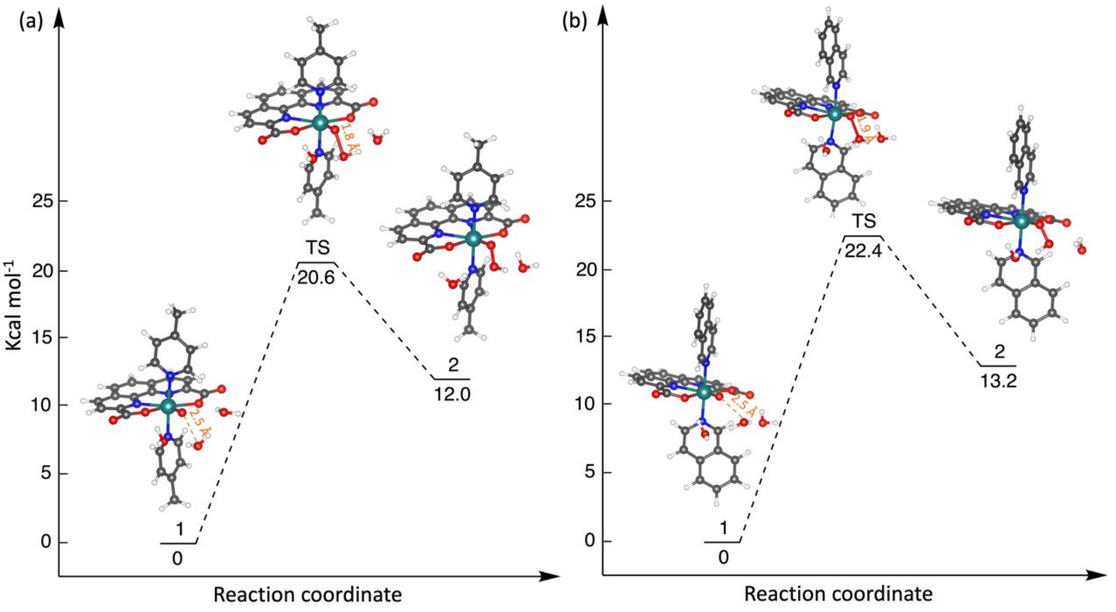

Figure S16 Free energy profile for the O-O bond formation of [Ru(pda)pic$_2$] (a) and [Ru(pda)isoq$_2$] (b) catalysts via the WNA reaction pathway. The snapshots presented in the profile are a configuration of a reactant (1), the transition state (TS), and a product (2) which were obtained from DFT simulations.

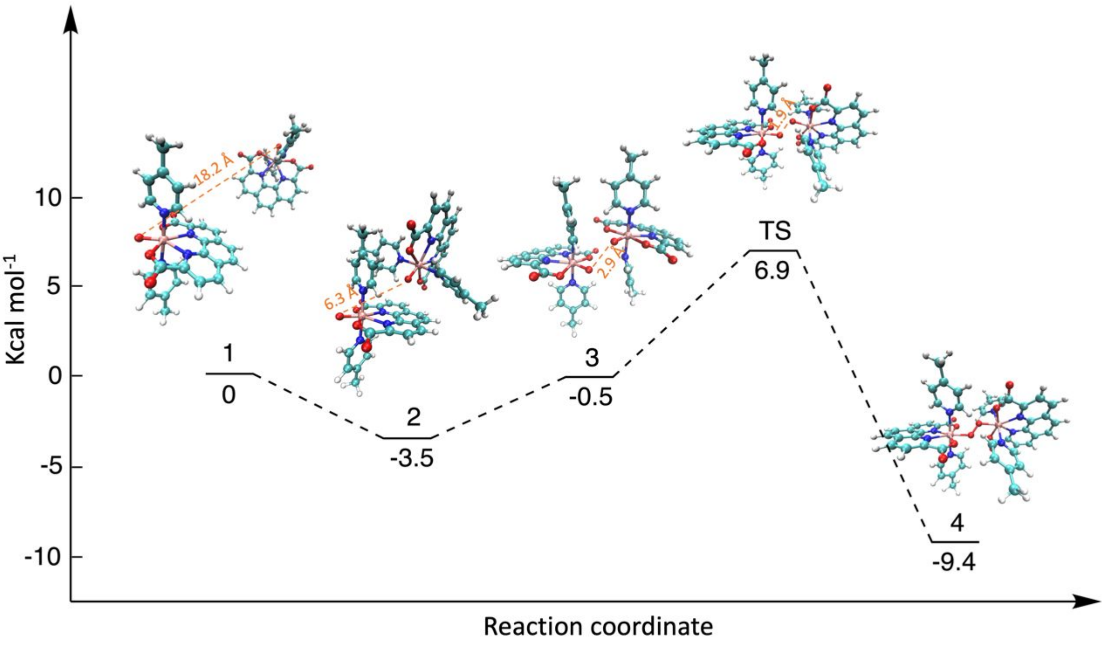

Figure S17 Free energy profile for the O-O bond formation of two [Ru(pda)pic$_2$] catalysts in the water phase. The snapshots presented in the profile are a configuration of two catalysts positioned relatively far apart (1), a front-to-back configuration (2), a prereactive dimer (3), the transition state (TS), and a product (4) which were obtained from umbrella sampling and EVB-MD simulations. The TIP3P water molecules have been omitted to enhance clarity.

Table S2 Activation free energy for forward (ΔΔG) and reverse (ΔΔG*) reaction of complexes Ru(bda)pic$_2$, Ru(bda)isoq$_2$, Ru(pda)pic$_2$, and Ru(pda)isoq$_2$. All values are in kcal mol^{-1}.

#	ΔΔG[a] gas	ΔΔG*[a] gas	ΔΔG[b] water	ΔΔG*[b] water	ΔG_{rearr}[c]	ΔG_{diss}
**Ru(bda)pic$_2$**	4.4	21.4	7.9 (6.9)	20.2 (20.4)	-	3.2
**Ru(bda)isoq$_2$**	7.9	24.8	7.1 (6.4)	28.9 (24.6)	-	6.3
**Ru(pda)pic$_2$**	5.2	20.0	7.4 (6.2)	16.3 (18.0)	3.0	3.5
**Ru(pda)isoq$_2$**	6.1	19.4	9.1 (6.3)	20.5 (18.5)		5.5

[a] All values calculated with DFT in the gas phase. [b] All values are calculated with EVB in the TIP3P water environment. DFT results with the PBF solvation model are provided in parenthesis for comparison. [c] Gibbs free energy needed to rearrange the complex to the correct pre-reactive complex from the front-to-back geometry.

Table S3 Atom types and partial charges assigned to atoms in the complex Ru(pda)isoq$_2$. The labelled atoms are shown in Figure S12.

Reactant Monomer	
**Atom type**	**Charge**
Ru1	0.339955
O2	-0.15247
C3	-0.146642
C4	0.098767
N5	-0.142433
O6	-0.453873
C7	0.589769
O8	-0.483817
C9	-0.09366
N10	-0.010156
C11	0.043551
C12	-0.303356
C13	-0.289691
C14	-0.095983
N15	0.070815
C16	-0.062483
C17	0.101317

C18	0.122855
C19	-0.19625
C20	-0.179975
C21	-0.00101
C22	0.143416
C23	-0.117775
C24	-0.130573
C25	0.03433
N26	-0.010861
C27	0.604191
O28	-0.455334
O29	-0.485121
C30	-0.096832
C31	0.276261
C32	0.092388
C33	-0.204432
C34	-0.093607
C35	-0.03644
C36	-0.266939
C37	0.269795
C38	0.095893
C39	-0.200677
C40	-0.078981
C41	-0.044633
C42	-0.263656
H43	0.175094
H44	0.146634
H45	0.147982
H46	0.193344
H47	0.182291
H48	0.154525
H49	0.165187
H50	0.17393
H51	0.171159
H52	0.167081
H53	0.176117
H54	0.163563
H55	0.140612
H56	0.141661
H57	0.136209
H58	0.172598
H59	0.164502

H60	0.139288
H61	0.138442
H62	0.164134

References

1. A. D. Bochevarov, E. Harder, T. F. Hughes, J. R. Greenwood, D. A. Braden, D. M. Philipp, D. Rinaldo, M. D. Halls, J. Zhang and R. A. Friesner, *International Journal of Quantum Chemistry*, 2013, **113**, 2110-2142.
2. Y. Zhao and D. G. Truhlar, *Theoretical chemistry accounts*, 2008, **120**, 215-241.
3. S. Grimme, J. Antony, S. Ehrlich and H. Krieg, *The Journal of chemical physics*, 2010, **132**, 154104.
4. L. Goerigk and S. Grimme, *Physical Chemistry Chemical Physics*, 2011, **13**, 6670-6688.
5. P. J. Hay and W. R. Wadt, *The Journal of chemical physics*, 1985, **82**, 299-310.
6. S. Zhan, B. Zhang, L. Sun and M. S. Ahlquist, *ACS Catalysis*, 2020, **10**, 13364-13370.
7. W. L. Jorgensen, D. S. Maxwell and J. Tirado-Rives, *Journal of the American Chemical Society*, 1996, **118**, 11225-11236.
8. H. J. Berendsen, D. van der Spoel and R. van Drunen, *Computer physics communications*, 1995, **91**, 43-56.
9. M. J. Abraham, T. Murtola, R. Schulz, S. Páll, J. C. Smith, B. Hess and E. Lindahl, *SoftwareX*, 2015, **1**, 19-25.
10. W. L. Jorgensen, J. Chandrasekhar, J. D. Madura, R. W. Impey and M. L. Klein, *The Journal of chemical physics*, 1983, **79**, 926-935.
11. U. Essmann, L. Perera, M. L. Berkowitz, T. Darden, H. Lee and L. G. Pedersen, *The Journal of chemical physics*, 1995, **103**, 8577-8593.
12. G. Bussi, D. Donadio and M. Parrinello, *The Journal of chemical physics*, 2007, **126**, 014101.
13. B. Hess, H. Bekker, H. J. Berendsen and J. G. Fraaije, *Journal of computational chemistry*, 1997, **18**, 1463-1472.
14. S. Kumar, J. M. Rosenberg, D. Bouzida, R. H. Swendsen and P. A. Kollman, *Journal of computational chemistry*, 1992, **13**, 1011-1021.
15. S. Zhan, D. Mårtensson, M. Purg, S. C. Kamerlin and M. S. Ahlquist, *Angewandte Chemie International Edition*, 2017, **56**, 6962-6965.
